# Nutrition and Lung Growth

**DOI:** 10.3390/nu10070919

**Published:** 2018-07-18

**Authors:** Michele Arigliani, Alessandro Mauro Spinelli, Ilaria Liguoro, Paola Cogo

**Affiliations:** Department of Medicine, University Hospital of Udine, Piazzale S. Maria Misericordia 1, 33100 Udine, Italy; alessandromspinelli@gmail.com (A.M.S.); ilarialiguoro@gmail.com (I.L.); paola.cogo@uniud.it (P.C.)

**Keywords:** lung development, lung function, intrauterine growth restriction malnutrition, vitamins, omega-3 fatty acids, pediatrics

## Abstract

Experimental evidence from animal models and epidemiology studies has demonstrated that nutrition affects lung development and may have a lifelong impact on respiratory health. Chronic restriction of nutrients and/or oxygen during pregnancy causes structural changes in the airways and parenchyma that may result in abnormal lung function, which is tracked throughout life. Inadequate nutritional management in very premature infants hampers lung growth and may be a contributing factor in the pathogenesis of bronchopulmonary dysplasia. Recent evidence seems to indicate that infant and childhood malnutrition does not determine lung function impairment even in the presence of reduced lung size due to delayed body growth. This review will focus on the effects of malnutrition occurring at critical time periods such as pregnancy, early life, and childhood, on lung growth and long-term lung function.

## 1. Introduction

Lung development is a multistage and multilevel process sustained by biochemical, mechanical and anatomical events spanning all gestational ages, from the end of the third week post-conception onwards, and continuing into post-natal life until around 22 years of age [1,2]. The lung has limited potential for recovery from early-life damage and poor lung function, a possible consequence of prenatal and perinatal insults, tracks throughout life with long-term consequences for respiratory health [3,4,5,6].

Nutrition has a key role in prenatal lung development, directly affecting mechanisms of lung growth but also influencing developmental programming through epigenetic changes [7,8]. The influence of nutrition on lung growth also continues in post-natal life, especially in early infancy [9].

Intrauterine growth restriction (IUGR) is the most common effect of chronic impaired prenatal nutrition, mostly (80–90%) due to reduced flow of nutrients and oxygen to the fetus through the placenta because of either placental insufficiency or maternal dietary deficiencies [10]. Placental insufficiency generally occurs in the second half of pregnancy, at the time of acinar and alveolar development, therefore the distal lung is most likely to be affected by IUGR [11,12,13].

In the neonatal period, adequate nutritional management is particularly important in extremely low birth weight infants (<1000 g), as growth failure in the first weeks of life is associated with an higher rate of bronchopulmonary dysplasia (BPD) in this group [14].

Less is known about the effects of infantile and childhood malnutrition on lung development. Recent evidence seems to indicate that it does not determine functional impairment but may affect lung size [15,16,17,18].

Micronutrients influence various aspects of the lung maturation. Retinoids from vitamin A regulate the expression of extracellular matrix proteins that address both airway development and alveolarization [19]. Vitamin D is involved in surfactant metabolism [20] and promotes epithelial–mesenchymal interactions that are very important for lung maturation [21]. Maternal deficiencies of vitamin E and selenium during pregnancy have been associated with an increased risk of wheezing episodes in offspring [22,23]. Finally, omega-3 fatty acids modulate inflammation and attenuate hyperoxic lung injury [12]; infants born at <30 weeks with low omega-3 plasma levels were found to be at higher risk of BPD [24].

This review will examine the effects of impaired nutrition and micronutrients deficit occurring during pregnancy, early life and childhood, on lung growth and respiratory health. Epidemiology aspects and pathophysiology mechanisms of the relationship between nutrition and the respiratory system will be discussed.

## 2. Fetal Nutrition and Lung Development

Our understanding of the effects of prenatal nutrition on lung growth comes mainly from experimental studies on animal models where conditions of utero-placental vascular insufficiency or maternal nutrient restriction (most commonly protein restriction) were reproduced at different stages of pregnancy. The most widely used animal models of IUGR are lambs, rats and mice. While in sheep the timing of lung development is very similar to that of humans, in rodents alveolarization occurs entirely after term birth and, thus, they represent an optimal model to evaluate the effects of IUGR without the complications of preterm birth [25]. However, the interpretation of these experimental studies is hampered by many factors, including heterogeneous experimental settings with few replication studies and the different animal species selected for the studies; thus, caution is needed when the findings of these studies are applied to humans [26].

On a macroscopic scale, lung growth is proportionally restricted to body growth in lambs that undergo prolonged fetal undernutrition induced by placental insufficiency [27].

Undernutrition during saccular and alveolar stage of lung development mainly impairs alveolarization. In sheep it leads to prenatal altered surfactant protein expression [28], decreased pulmonary vascular growth [29], post-natal permanent reduction of alveolar surface area in relation to lung volume [30,31], increased extracellular matrix, and a thickened blood-gas barrier [31,32], resulting in reduced pulmonary compliance and diffusing capacity [33]. Rodent models of IUGR, at the saccular stage of lung development show thickened distal air space walls [34], and decreased elastin expression and elastic fiber deposition in the extracellular matrix [35].

Evidence from animal models suggests that IUGR also affects the conductive airways. In near-term growth-restricted lambs’ fetuses, it was shown that luminal areas, basement membrane perimeters, and airway wall sections of the trachea and larger bronchi have a smaller size [36], while the mucosa showed reduced folding and sparse ciliated epithelial cells [37].

Epigenetic changes induced by chronic fetal undernutrition and IUGR are likely to be responsible for most of these effects [25], impacting signaling pathways that are involved in lung maturation, such as the peroxisome proliferator-activated receptor (PPAR) pathway [38,39] or transforming growth factor (TGF-β) signaling [40]. Of note, undernutrition may not be the only cause of altered developmental programming of the lung. Recent experimental data showed that in mice prenatal exposure to conditions commonly associated with maternal overfeeding in the western world (high-fat diet and hyperglycemia) caused feto-placental inflammation and ultimately delayed fetal lung development, alveolar simplification, and altered nitric oxide metabolism in the airways [41,42], whereas in lambs late gestation maternal overnutrition was associated with impaired surfactant production [43].

## 3. Effects of IUGR on Lung Function and Respiratory Health

There is conflicting evidence as to whether IUGR increases [44,45,46], has no impact [47,48,49], or even decreases [50,51,52] the incidence of neonatal respiratory distress syndrome. Conversely it is well recognized that IUGR is associated with an increased risk of BPD in preterm infants born before 32 weeks of gestational age [53,54,55,56,57].

A few studies investigated the impact of IUGR on subsequent lung function and wheezing or asthma. A population-based prospective cohort study (“Generation R”) involving over 5000 children in the Netherlands found that IUGR was associated with higher respiratory resistance in 6-year old children [58], while fetal growth pattern was not associated with wheezing until the age of 3 years [59] or 4 years [60] according to different birth cohort studies. Turner et al. found that low fetal growth during the second and third trimesters was associated with reduced Forced expiratory volume in 1st second (FEV_1_) and Forced Vital Capacity (FVC) but not altered asthma risk at 10 years of age [61]. Data from the “Generation R” cohort also indicated that restricted fetal weight growth during the second and third trimesters (weight growth percentile change between the time periods <−0.67 z-scores) was associated with lower dynamic lung volumes at age 10 years, partly dependent upon infant weight growth patterns, whereas there was no association with asthma or recurrent wheezing [62]. Rapid weight gain in infancy in children with IUGR was associated in some studies with increased risk of wheezing [60,63], asthma [62,63], and abnormal lung function in later life [64]. There is also evidence that IUGR may exacerbate the negative impact of prematurity on lung function in later life [65,66].

Reduced lung function was shown in infants [67,68,69] and children [66,70] with low birth weight for gestation (below the 10th percentile, a proxy for IUGR) even in the presence of catch-up growth [70]. A metanalysis of 24 birth cohort studies [71], showed that low birth weight and greater infant weight gain, normalized as z-scores, are associated with an increased risk of childhood asthma, lower FEV_1_ and FEV_1_/FVC z-score.

These findings could be the consequence of dysynaptic lung growth [72]. After birth, airways increase only in size whereas alveoli continue to increase in both size and number [73], therefore lung parenchyma has higher potential than airways to compensate prenatal impairment, as indicated by evidence of catch-up alveolarization in former preterm children [74,75]. A disproportionate growth between lung parenchyma and airways size may determine airflow limitation [76] and might contribute to the finding of abnormal lung function (mostly impaired FEV_1_ and FEV_1_/FVC) in some children with restricted fetal growth [61,62,71].

As young adults, subjects with low birth weight may reach a poor maximal lung function [77,78,79], which is associated with an increased risk of developing chronic obstructive pulmonary disease (COPD) in late adult life [80].

A possible link between fetal malnutrition and COPD was also suggested by the increased prevalence of obstructive respiratory disease in people aged about 50, born to mothers who had been exposed to the Dutch famine in the Netherlands (1944–1945). Controls of similar age born before or conceived after the famine were also included. In particular, subjects exposed to famine at mid-gestation showed the greatest effect (odds ratio—OR—adjusted 1.7, 95% CI, 1.1 to 2.6) [81].

## 4. Postnatal Early Nutrition and Lung Growth

After birth, nutrition plays a critical role for respiratory development of premature born infants, especially for very low birth weight (VLBW) infants whose saccular-alveolar stage of lung maturation occurs mostly or entirely in post-natal life [11,25].

The effects of post-natal undernutrition on lung maturation have been characterized in animal models. Rodent models, which are at a saccular stage of lung development when born at term [7], suggest that early post-natal undernutrition disrupts alveolarization [82] and affects the bronchiolar epithelium [83], with a reduction of both epithelial cells division and conversion of Clara cells to ciliated cells. Early post-natal nutritional restriction in rabbits exacerbates the negative impact of hyperoxia on alveolarization and extra-cellular matrix deposition in the lung [84,85], whereas in piglets it affects the oxidative capacity and the expression of myosin heavy chain isoforms in the diaphragm [86].

To what extent these experimental findings can be translated to humans is not known but there is some evidence indicating that nutritional management of prematurely born infants may be involved in the pathogenesis of BPD [87]. A large multicenter study showed that VLBW infants that grew at the lowest quartile in the first three weeks of life were at higher risk for BPD and neurodevelopmental problems [14]. Excessive fluid intake also might play a role: in a cohort of nearly 1400 extremely low birth weight infants (birth weight 401 to 1000 g), infants who survived without BPD had significantly lower average total fluid intake (parenteral and enteral) from the second to the 10th day of life (*p* < 0.001) and significantly higher average weight loss (expressed as percent of birth weight) between days 6–9 (*p* < 0.05) compared to infants who died or developed BPD [88]. A systematic review of five studies evaluating the impact of restricted versus liberal water intake in preterm infants, pointed to a trend toward reduced risk of BPD in infants undergoing fluid restriction, although the five trials were not homogeneous in terms of study population, duration of the intervention, and targeted volume intakes [89]. Moreover, fluid restriction in preterm infants has to be balanced with the need for adequate caloric intake, as poor nutrition may be a determinant of BPD [90]. A trial comparing early versus delayed sodium supplementation (4 mmol/kg/day started on either the second day of life or when weight loss was 6% of birthweight) in preterm infants of 25–30 weeks of gestational age, did not find significant differences in the rate of continuing oxygen requirement at 28 days of life [91].

In infants with evolving or established BPD, nutritional management is also very important as they often show growth failure [90]. However nutritional management of these infants lies beyond the purpose of this review and has been discussed elsewhere [87], while the influence of micronutrients on post-natal lung growth in early life will be discussed later.

Breastfeeding seems to have a protective role against viral wheezing, especially in the first two years of life [92]. This effect is probably mediated by immune system-enhancing properties of human breast milk that limit the rate of viral respiratory infections in infancy [93,94,95]. In contrast, the impact of breastfeeding on later childhood asthma remains unclear [92,96]. The relationship between breastfeeding and subsequent lung function has been also largely investigated: a systematic review of five birth cohorts and three cross-sectional studies [97] found that children and adolescents who had been breastfed for more than 4 months had a 20–100 mL greater FVC than children who received formula feeding. A positive but weaker association between breastfeeding for more than 4 months and FEV_1_ was also reported in some longitudinal studies [98,99], but not in others [100,101,102]. More recently, spirometry data recorded at 10 years of age in over 4000 children who had been breastfed showed that breastfeeding shorter than six months was associated with lower FEV_1_ and FVC, although the impact was limited (z-score change for both FEV_1_ and FVC, −0.01, 95% CI, −0.02 to 0.00; per month shorter breastfeeding) and probably clinically not relevant [103]. Overall, these findings suggest that breastfeeding might enhance lung growth, possibly through the action of cytokines and growth factors involved in lung maturation, such as TGF-β [104], or inducing epigenetic changes [105].

## 5. Malnutrition and Lung Growth in Childhood

The effects of infant and childhood malnutrition on lung development have been poorly investigated so far. This field of research could be particularly relevant for low- and middle-income countries due to the high burden of malnutrition and respiratory disease in such settings.

Alveolarization continues until at least 2 years of age [2], and therefore infant malnutrition could be expected to have greater long-term consequences on lung development compared to undernutrition occurring later in childhood. A recent study by Lelijveld et al. [106] investigated spirometry outcomes in a cohort (“ChroSAM study”) of Malawian children, 7 years after a hospital admission for severe acute malnutrition (SAM). Siblings and healthy children from the same community were also evaluated. Surprisingly, 237 survivors of SAM had lung function comparable to the two control groups (143 siblings and 121 community controls), although it should be noted that 46% of the case group died before follow-up, representing a group which probably included the most severe SAM cases and could have had poorer lung function. Measuring sitting height, a pattern of shorter legs but preserved torso height was found in SAM survivors that might allow lung function to be preserved. Children who had suffered from SAM before two years of age (a period of greater susceptibility of lung development toward external influences) did not differ from the rest of the case group [106]. In a cross-sectional study including over 1000 school-age children from different parts of sub-Saharan Africa [17], it was shown that undernourished children (Body Mass Index—BMI—z-score < −2) had reduced FEV_1_ and FVC z-scores compared to normotrophic children but a preserved FEV_1_/FVC ratio, implying smaller chest diameters without evidence of functional impairment. Also in that study, children with the lowest BMI z-scores had relatively shorter legs compared to torso height (highest sitting/standing height ratio), as previously found in SAM survivors. A secondary analysis of the ChroSAM cohort [18] confirmed that children with a BMI z-score < −2 have proportionally reduced dynamic lung volumes with normal FEV_1_/FVC ratio z-score. Similar findings were also previously reported by Sonappa et al. in semi-urban and rural Indian children [16], that had significant reductions of ~0.5–0.9 z-scores in both FEV_1_ and FVC (with preserved FEV_1_/FVC ratio) and of ~1 z-score in BMI, as compared to Indian urban counterparts and children of Indian ancestry living in the UK. Overall these studies seem to support the hypothesis of a “lung-sparing growth” in children with malnutrition, where lung development is relatively spared compared to other less vital organs [107].

## 6. Influence of Micronutrients on Lung Development

Micronutrient deficiency is common worldwide and vitamins are extensively recognized as being important for the developing fetus and neonate [108]. Pregnant women have higher metabolic demands and are at risk of micronutrient deficiency [109], especially those of low socio-economic status from developing countries [110,111].

Micronutrients with the most relevant effects on lung development are Vitamins A, D, E, selenium and omega-3 docosahexaenoic acid.

### 6.1. Vitamin A

Biologically active metabolites of Vitamin A (e.g., all-trans retinoic acid) are involved in many aspects of lung development. In mice, retinoic acid contributes to branching morphogenesis and to limiting excess branching and smooth muscle proliferation in the airways [112,113]. Retinoids are also important for adequate formation and maintenance of the alveoli [114,115] and have a role in regulating type II pneumocyte proliferation and synthesis of surfactant-associated proteins B and C (SP-B and SP-C, respectively) [116]. Many of these effects depend on the modulating activity of retinoids on the expression of several extracellular matrix proteins, most importantly elastin [117,118].

With regard to the effects of vitamin A supplementation on lung development, in animal models with prenatal vitamin A-deficiency, subsequent treatment with retinoic acid reversed some of the structural alterations that had been induced by missing retinol activity [119,120]. Vitamin A supplementation in very low birthweight infants slightly decreases the incidence of BPD [121,122], but does not affect duration of mechanical ventilation, length of hospital stay, and neurodevelopmental outcomes at 18–22 months of age [123]. Long-term effects of vitamin A supplementation in pregnant women were documented in a region with endemic vitamin A deficiency, where children whose mothers received vitamin A supplementation until 6 months after pregnancy had better lung function at 9 to 11 years of age than children whose mothers had received beta carotene supplementation or placebo [124].

### 6.2. Vitamin D

The importance of vitamin D for fetal lung development was shown in animal models in several studies. Vitamin D contributes to pneumocyte type II maturation [125], enhances biosynthesis of SP-B [20] and surfactant-related phospholipids [124], and stimulates surfactant release [126]. Moreover, it promotes alveolar epithelial-mesenchymal interactions during lung maturation [127]. Prenatal vitamin D deficiency determines increased airway smooth muscle mass and airway hyper-responsiveness in rats [128], whereas in a mouse model it was associated with increased airway resistance and decreased lung volumes [129]. Genomic analyses in lung tissue from mice and humans have identified several genes involved in lung development that are regulated by vitamin D [130].

Moving to human studies, maternal vitamin D deficiency during pregnancy and low levels of 25-hydroxyvitamin D at birth were associated with decreased lung function in offspring by the age of 6 years [131,132]. A recent metanalysis of birth cohort studies that measured vitamin D blood levels in pregnant women or in cord blood at birth showed a borderline positive association between prenatal circulating 25-hydroxyvitamin D levels and FEV_1_ of offspring at school age (FEV_1_ z-score coefficient 0.07, 95% CI, −0.01 to 0.15), but no significant association with wheezing or asthma [133]. The combined analysis of two independent trials [134,135] that randomized pregnant women to receive high-dose vitamin D3 supplementation (4000 IU/d or 2400 IU/d,) versus 400 IU/d (controls), showed that children whose mothers received high-dose vitamin D3 had a 25% reduced risk of recurrent wheezing by the age of 3 years (adjusted OR, 0.74, 95% CI, 0.57 to 0.96, *p* = 0.02) [136]. These data were recently confirmed by a systematic review indicating that maternal vitamin D supplementation during pregnancy consistently reduces the risk of wheezing in children by the age of 3 years (risk ratio 0.81, 95% CI, 0.67 to 0.98) [137].

### 6.3. Vitamin E

Vitamin E it is not synthesized by the human body and requires intake from food sources, typically oils. There are eight isoforms of Vitamin E, most studies evaluating α-tocopherol and γ-tocopherol. Maternal plasma level of vitamin E during pregnancy are positively associated with fetal growth [138]. In a birth cohort, body length of human fetuses (crown–rump length) in the first trimester of pregnancy was positively associated with maternal plasma α-tocopherol, and with FEV_1_ and FVC at 5 years of age [139], leading the authors to hypothesize that vitamin E may enhance fetal lung growth. A metanalysis of seven birth cohort studies found that higher maternal dietary vitamin E intake was associated with a 46% reduction in the odds of wheezing during childhood (pooled OR 0.54, 95% CI, 0.41 to 0.71) while there was no association with asthma [140]. Vitamin E is an antioxidant and has a protective role against oxygen toxicity [141]. In premature infants with respiratory distress syndrome, lower total plasma levels of vitamin E and selenium were associated with increased risk of developing BPD [142,143]. Unfortunately, trials of high-dose vitamin E (α-tocopherol) supplementation in very preterm born infant to prevent BPD showed variable results [144] and an association with increased risk of sepsis [145].

### 6.4. Selenium

Selenium acts together with vitamin E to prevent peroxide formation [9]. In rats selenium deficiency impairs lung development, in particular alveolar septation [146]. A significant negative association was found between maternal plasma selenium concentration during pregnancy and the risk of wheezing episodes in offspring in the first 3 years of life, with no association with asthma [23,147]. In very low birthweight infants, low plasma selenium level is significantly associated with increased respiratory morbidity [148], however selenium supplementation did not have any impact on the incidence of BPD [149].

### 6.5. Zinc

There is some evidence of abnormal prenatal lung development resulting from maternal zinc deficiency in rats [150]. Maternal zinc intake during pregnancy in the highest quartile was associated with a reduction in the odds of wheezing episodes in preschool children in three birth cohort studies [151,152,153] (pooled OR, 0.57, 95% CI, 0.40 to 0.81) [153], whereas no association resulted from other studies [22,154,155].

### 6.6. Docosahexaenoic Acid

Docosahexaenoic acid (DHA) is a n-3 long-chain polyunsaturated fatty acid (LCPUFA) that enhances lung maturation and modulates inflammation [12,156]. In rodent models, maternal supplementation of DHA during late gestation and lactation increases surfactant concentration in amniotic fluid and in the fetal lung [157], enhances alveolarization [158], and ameliorates lung alterations due to IUGR [159]. Moreover, DHA supplementation decreases inflammation and attenuates lung injury induced by hyperoxia [160,161,162,163,164]. Premature infants are typically deficient in DHA [165] and those delivered at the lowest gestational ages are at the highest risk of deficiency [166]. In infants born at <30 weeks, low DHA levels were associated with a 2.5 times increased risk (OR, 2.5, 95% CI, 1.3 to 5.0) of BPD [24]. Based on evidence that DHA modulates inflammation and lung injury due to hyperoxia in animal models, some trials evaluated the utility of DHA supplementation in preterm-born infants for preventing BPD [167,168,169]. The most recent and best designed study [169] randomized 1205 preterm infants born before 29 weeks to receive an emulsion containing 60 mg/kg of DHA or a control (soy) emulsion, started within 3 days after the first enteral feeding and continued until 36 weeks of postmenstrual age. No significant difference in the incidence of BPD between the two groups (53.2% in the study group vs. 49.7% in controls, *p* 0.06) was reported.

With regard to the long-term effect of maternal n-3 LCPUFA supplementation in pregnancy, Bisgaard et al. recently reported a 30% relative reduction in the probability of persistent wheezing at 3 years of age and asthma at 5 years in children whose mothers were supplemented with fish oil (2.4 g of n-3 LCPUFA per day) during pregnancy [170].

## 7. Conclusions

Experimental evidence and epidemiology studies show that impaired nutrition may have many possible consequences for lung maturation, depending on the type of nutritional deficit and the stage of lung development at which it occurs. In particular, malnutrition during late fetal life mainly affects the distal lung, exacerbates the negative effects of prematurity, and may determine life-long lung function impairment, probably increasing the susceptibility to COPD in later adult life. Suboptimal nutritional management in preterm infants may contribute to growth failure in the first weeks of life, which has been associated with an increased incidence of BPD. Micronutrients also play an important role in prenatal lung growth and their deficit in critical phases of lung development may have an impact on respiratory morbidity in preterm infants and on the incidence of wheezing in later life. Breastfeeding seems to be associated with lower incidence of preschool wheezing and better lung function at school age. Finally childhood malnutrition might be associated with a “lung-sparing growth” pattern which preserves normal lung function even in the presence of smaller lung size.

Overall many aspects of the effects of malnutrition on lung growth need to be elucidated. The most important are the reproducibility in humans of several physiopathology mechanisms demonstrated in animal models, or the best strategies for micronutrient supplementation during pregnancy and in preterm infants, and, finally, the relationship between IUGR and COPD. The last point could be addressed only by studying the incidence of COPD in people with a certain prenatal diagnosis of IUGR and in relation to the physio-pathological mechanism that induces IUGR. Future studies should address these points.

## References

[1] Stocks J., Hislop A., Sonnappa S. (2013). Early lung development: Lifelong effect on respiratory health and disease. Lancet Respir. Med..

[2] Burri P.H. (2006). Structural Aspects of Postnatal Lung Development–Alveolar Formation and Growth. Neonatology.

[3] Berry C.E., Billheimer D., Jenkins I.C., Lu Z.J., Stern D.A., Gerald L.B., Carr T.F., Guerra S., Morgan W.J., Wright A.L. (2016). A Distinct Low Lung Function Trajectory from Childhood to the Fourth Decade of Life. Am. J. Respir. Crit. Care Med..

[4] Belgrave D.C.M., Granell R., Turner S.W., Curtin J.A., Buchan I.E., Le Souëf P.N., Simpson A., Henderson A.J., Custovic A. (2018). Lung function trajectories from pre-school age to adulthood and their associations with early life factors: A retrospective analysis of three population-based birth cohort studies. Lancet Respir. Med..

[5] Bui D.S., Lodge C.J., Burgess J.A., Lowe A.J., Perret J., Bui M.Q., Bowatte G., Gurrin L., Johns D.P., Thompson B.R. (2018). Childhood predictors of lung function trajectories and future COPD risk: A prospective cohort study from the first to the sixth decade of life. Lancet Respir. Med..

[6] Bush A. (2016). Lung Development and Aging. Ann. Am. Thorac. Soc..

[7] Joss-Moore L.A., Albertine K.H., Lane R.H. (2011). Epigenetics and the developmental origins of lung disease. Mol. Genet. Metab..

[8] Harding R., Maritz G. (2012). Maternal and fetal origins of lung disease in adulthood. Semin. Fetal. Neonatal Med..

[9] Harding R., De Matteo R. (2014). Chapter 19—The Influence of Nutrition on Lung Development before and after Birth. The Lung.

[10] Sharma D., Shastri S., Sharma P. (2016). Intrauterine Growth Restriction: Antenatal and Postnatal Aspects. Clin. Med. Insights Pediatr..

[11] Briana D.D., Malamitsi-Puchner A. (2013). Small for gestational age birth weight: Impact on lung structure and function. Paediatr. Respir. Rev..

[12] Moya F. (2014). Preterm Nutrition and the Lung. Nutr. Care Preterm Infants.

[13] Pike K., Jane Pillow J., Lucas J.S. (2012). Long term respiratory consequences of intrauterine growth restriction. Semin. Fetal. Neonatal Med..

[14] Ehrenkranz R.A., Dusick A.M., Vohr B.R., Wright L.L., Wrage L.A., Poole W.K. (2006). Growth in the neonatal intensive care unit influences neurodevelopmental and growth outcomes of extremely low birth weight infants. Pediatrics.

[15] Quanjer P.H. (2015). Lung function, genetics and socioeconomic conditions. Eur. Respir. J..

[16] Sonnappa S., Lum S., Kirkby J., Bonner R., Wade A., Subramanya V., Lakshman P.T., Rajan B., Nooyi S.C., Stocks J. (2015). Disparities in Pulmonary Function in Healthy Children across the Indian Urban–Rural Continuum. Am. J. Respir. Crit. Care Med..

[17] Arigliani M., Canciani M.C., Mottini G., Altomare M., Magnolato A., Loa Clemente S.V., Tshilolo L., Cogo P., Quanjer P.H. (2017). Evaluation of the Global Lung Initiative 2012 Reference Values for Spirometry in African Children. Am. J. Respir. Crit. Care Med..

[18] Lelijveld N., Kirkby J. (2018). Spirometry in undernourished children in sub-Saharan African. J. Public Health Emerg..

[19] Massaro D., Massaro G.D. (2010). Lung development, lung function, and retinoids. N. Engl. J. Med..

[20] Chen L., Wilson R., Bennett E., Zosky G.R. (2016). Identification of vitamin D sensitive pathways during lung development. Respir. Res..

[21] Lykkedegn S., Sorensen G.L., Beck-Nielsen S.S., Pilecki B., Duelund L., Marcussen N., Christesen H.T. (2016). Vitamin D Depletion in Pregnancy Decreases Survival Time, Oxygen Saturation, Lung Weight and Body Weight in Preterm Rat Offspring. PLoS ONE.

[22] Devereux G., Turner S.W., Craig L.C.A., McNeill G., Martindale S., Harbour P.J., Helms P.J., Seaton A. (2006). Low maternal vitamin E intake during pregnancy is associated with asthma in 5-year-old children. Am. J. Respir. Crit. Care Med..

[23] Devereux G., McNeill G., Newman G., Turner S., Craig L., Martindale S., Helms P., Seaton A. (2007). Early childhood wheezing symptoms in relation to plasma selenium in pregnant mothers and neonates. Clin. Exp. Allergy J. Br. Soc. Allergy Clin. Immunol..

[24] Martin C.R., Dasilva D.A., Cluette-Brown J.E., Dimonda C., Hamill A., Bhutta A.Q., Coronel E., Wilschanski M., Stephens A.J., Driscoll D.F. (2011). Decreased postnatal docosahexaenoic and arachidonic acid blood levels in premature infants are associated with neonatal morbidities. J. Pediatr..

[25] Joss-Moore L.A., Lane R.H., Albertine K.H. (2015). Epigenetic Contributions to the Developmental Origins of Adult Lung Disease. Biochem. Cell Biol. Biochim. Biol. Cell..

[26] Swanson A.M., David A.L. (2015). Animal models of fetal growth restriction: Considerations for translational medicine. Placenta.

[27] Cock M.L., Albuquerque C.A., Joyce B.J., Hooper S.B., Harding R. (2001). Effects of intrauterine growth restriction on lung liquid dynamics and lung development in fetal sheep. Am. J. Obstet. Gynecol..

[28] Orgeig S., Crittenden T.A., Marchant C., McMillen I.C., Morrison J.L. (2010). Intrauterine growth restriction delays surfactant protein maturation in the sheep fetus. Am. J. Physiol. Lung Cell. Mol. Physiol..

[29] Rozance P.J., Seedorf G.J., Brown A., Roe G., O’Meara M.C., Gien J., Tang J.-R., Abman S.H. (2011). Intrauterine growth restriction decreases pulmonary alveolar and vessel growth and causes pulmonary artery endothelial cell dysfunction in vitro in fetal sheep. Am. J. Physiol. Lung Cell. Mol. Physiol..

[30] Lipsett J., Tamblyn M., Madigan K., Roberts P., Cool J.C., Runciman S.I.C., McMillen I.C., Robinson J., Owens J.A. (2006). Restricted fetal growth and lung development: A morphometric analysis of pulmonary structure. Pediatr. Pulmonol..

[31] Maritz G.S., Cock M.L., Louey S., Joyce B.J., Albuquerque C.A., Harding R. (2001). Effects of fetal growth restriction on lung development before and after birth: A morphometric analysis. Pediatr. Pulmonol..

[32] Maritz G.S., Cock M.L., Louey S., Suzuki K., Harding R. (2004). Fetal growth restriction has long-term effects on postnatal lung structure in sheep. Pediatr. Res..

[33] Joyce B.J., Louey S., Davey M.G., Cock M.L., Hooper S.B., Harding R. (2001). Compromised respiratory function in postnatal lambs after placental insufficiency and intrauterine growth restriction. Pediatr. Res..

[34] O’Brien E.A., Barnes V., Zhao L., McKnight R.A., Yu X., Callaway C.W., Wang L., Sun J.C., Dahl M.J., Wint A. (2007). Uteroplacental insufficiency decreases p53 serine-15 phosphorylation in term IUGR rat lungs. Am. J. Physiol. Regul. Integr. Comp. Physiol..

[35] Joss-Moore L.A., Wang Y., Yu X., Campbell M.S., Callaway C.W., McKnight R.A., Wint A., Dahl M.J., Dull R.O., Albertine K.H. (2011). IUGR decreases elastin mRNA expression in the developing rat lung and alters elastin content and lung compliance in the mature rat lung. Physiol. Genom..

[36] Wignarajah D., Cock M.L., Pinkerton K.E., Harding R. (2002). Influence of intrauterine growth restriction on airway development in fetal and postnatal sheep. Pediatr. Res..

[37] Rees S., Ng J., Dickson K., Nicholas T., Harding R. (1991). Growth retardation and the development of the respiratory system in fetal sheep. Early Hum. Dev..

[38] Joss-Moore L.A., Wang Y., Ogata E.M., Sainz A.J., Yu X., Callaway C.W., McKnight R.A., Albertine K.H., Lane R.H. (2011). IUGR differentially alters MeCP2 expression and H3K9Me3 of the PPARγ gene in male and female rat lungs during alveolarization. Birt. Defects Res. A Clin. Mol. Teratol..

[39] Zana-Taieb E., Pham H., Franco-Montoya M.L., Jacques S., Letourneur F., Baud O., Jarreau P.H., Vaiman D. (2015). Impaired alveolarization and intra-uterine growth restriction in rats: A postnatal genome-wide analysis. J. Pathol..

[40] Alejandre Alcázar M.A., Morty R.E., Lendzian L., Vohlen C., Oestreicher I., Plank C., Schneider H., Dötsch J. (2011). Inhibition of TGF-β Signaling and Decreased Apoptosis in IUGR-Associated Lung Disease in Rats. PLoS ONE.

[41] Mayor R.S., Finch K.E., Zehr J., Morselli E., Neinast M.D., Frank A.P., Hahner L.D., Wang J., Rakheja D., Palmer B.F. (2015). Maternal high-fat diet is associated with impaired fetal lung development. Am. J. Physiol.-Lung Cell. Mol. Physiol..

[42] Grasemann C., Herrmann R., Starschinova J., Gertsen M., Palmert M.R., Grasemann H. (2017). Effects of fetal exposure to high-fat diet or maternal hyperglycemia on L-arginine and nitric oxide metabolism in lung. Nutr. Diabetes.

[43] Lock M.C., McGillick E.V., Orgeig S., McMillen I.C., Mühlhäusler B.S., Zhang S., Morrison J.L. (2017). Differential effects of late gestation maternal overnutrition on the regulation of surfactant maturation in fetal and postnatal life. J. Physiol..

[44] Zaw W., Gagnon R., da Silva O. (2003). The risks of adverse neonatal outcome among preterm small for gestational age infants according to neonatal versus fetal growth standards. Pediatrics.

[45] Qiu X., Lodha A., Shah P.S., Sankaran K., Seshia M.M.K., Yee W., Jefferies A., Lee S.K. (2012). Canadian Neonatal Network Neonatal outcomes of small for gestational age preterm infants in Canada. Am. J. Perinatol..

[46] Engineer N., Kumar S. (2010). Perinatal variables and neonatal outcomes in severely growth restricted preterm fetuses. Acta Obstet. Gynecol. Scand..

[47] Nobile S., Marchionni P., Carnielli V.P. (2017). Neonatal outcome of small for gestational age preterm infants. Eur. J. Pediatr..

[48] Tsai L.-Y., Chen Y.-L., Tsou K.-I., Mu S.-C., Taiwan Premature Infant Developmental Collaborative Study Group (2015). The impact of small-for-gestational-age on neonatal outcome among very-low-birth-weight infants. Pediatr. Neonatol..

[49] Giapros V., Drougia A., Krallis N., Theocharis P., Andronikou S. (2012). Morbidity and mortality patterns in small-for-gestational age infants born preterm. J. Matern.-Fetal Neonatal Med..

[50] De Jesus L.C., Pappas A., Shankaran S., Li L., Das A., Bell E.F., Stoll B.J., Laptook A.R., Walsh M.C., Hale E.C. (2013). Outcomes of small for gestational age infants born at <27 weeks’ gestation. J. Pediatr..

[51] Bartels D.B., Kreienbrock L., Dammann O., Wenzlaff P., Poets C.F. (2005). Population based study on the outcome of small for gestational age newborns. Arch. Dis. Child. Fetal Neonatal Ed..

[52] Sharma P., McKay K., Rosenkrantz T.S., Hussain N. (2004). Comparisons of mortality and pre-discharge respiratory outcomes in small-for-gestational-age and appropriate-for-gestational-age premature infants. BMC Pediatr..

[53] Morrow L.A., Wagner B.D., Ingram D.A., Poindexter B.B., Schibler K., Cotten C.M., Dagle J., Sontag M.K., Mourani P.M., Abman S.H. (2017). Antenatal Determinants of Bronchopulmonary Dysplasia and Late Respiratory Disease in Preterm Infants. Am. J. Respir. Crit. Care Med..

[54] Jensen E.A., Foglia E.E., Dysart K.C., Simmons R.A., Aghai Z.H., Cook A., Greenspan J.S., DeMauro S.B. (2018). Adverse effects of small for gestational age differ by gestational week among very preterm infants. Arch. Dis. Child. Fetal Neonatal Ed..

[55] Eriksson L., Haglund B., Odlind V., Altman M., Ewald U., Kieler H. (2015). Perinatal conditions related to growth restriction and inflammation are associated with an increased risk of bronchopulmonary dysplasia. Acta Paediatr. Oslo Nor. 1992.

[56] Bose C., Van Marter L.J., Laughon M., O’Shea T.M., Allred E.N., Karna P., Ehrenkranz R.A., Boggess K., Leviton A., Extremely Low Gestational Age Newborn Study Investigators (2009). Fetal growth restriction and chronic lung disease among infants born before the 28th week of gestation. Pediatrics.

[57] Henderson-Smart D.J., Hutchinson J.L., Donoghue D.A., Evans N.J., Simpson J.M., Wright I., Australian and New Zealand Neonatal Network (2006). Prenatal predictors of chronic lung disease in very preterm infants. Arch. Dis. Child. Fetal Neonatal Ed..

[58] Sonnenschein-van der Voort A.M.M., Gaillard R., de Jongste J.C., Hofman A., Jaddoe V.W.V., Duijts L. (2016). Foetal and infant growth patterns, airway resistance and school-age asthma. Respirology.

[59] Pike K.C., Crozier S.R., Lucas J.S.A., Inskip H.M., Robinson S., Roberts G., Godfrey K.M., Southampton Women’s Survey Study Group (2010). Patterns of fetal and infant growth are related to atopy and wheezing disorders at age 3 years. Thorax.

[60] Sonnenschein-van der Voort A.M.M., Jaddoe V.W.V., Raat H., Moll H.A., Hofman A., de Jongste J.C., Duijts L. (2012). Fetal and infant growth and asthma symptoms in preschool children: The Generation R Study. Am. J. Respir. Crit. Care Med..

[61] Turner S., Prabhu N., Danielan P., McNeill G., Craig L., Allan K., Cutts R., Helms P., Seaton A., Devereux G. (2011). First- and second-trimester fetal size and asthma outcomes at age 10 years. Am. J. Respir. Crit. Care Med..

[62] den Dekker H.T., Jaddoe V.W.V., Reiss I.K., de Jongste J.C., Duijts L. (2017). Fetal and Infant Growth Patterns and Risk of Lower Lung Function and Asthma. The Generation R Study. Am. J. Respir. Crit. Care Med..

[63] Sonnenschein-van der Voort A.M.M., Arends L.R., de Jongste J.C., Annesi-Maesano I., Arshad S.H., Barros H., Basterrechea M., Bisgaard H., Chatzi L., Corpeleijn E. (2014). Preterm birth, infant weight gain, and childhood asthma risk: A meta-analysis of 147,000 European children. J. Allergy Clin. Immunol..

[64] van der Gugten A.C., Koopman M., Evelein A.M.V., Verheij T.J.M., Uiterwaal C.S.P.M., van der Ent C.K. (2012). Rapid early weight gain is associated with wheeze and reduced lung function in childhood. Eur. Respir. J..

[65] Greenough A., Yuksel B., Cheeseman P. (2004). Effect of in utero growth retardation on lung function at follow-up of prematurely born infants. Eur. Respir. J..

[66] Ronkainen E., Dunder T., Kaukola T., Marttila R., Hallman M. (2016). Intrauterine growth restriction predicts lower lung function at school age in children born very preterm. Arch. Dis. Child. Fetal Neonatal Ed..

[67] Hoo A.-F., Stocks J., Lum S., Wade A.M., Castle R.A., Costeloe K.L., Dezateux C. (2004). Development of Lung Function in Early Life. Am. J. Respir. Crit. Care Med..

[68] Dezateux C., Lum S., Hoo A.-F., Hawdon J., Costeloe K., Stocks J. (2004). Low birth weight for gestation and airway function in infancy: Exploring the fetal origins hypothesis. Thorax.

[69] Gray D., Willemse L., Visagie A., Czövek D., Nduru P., Vanker A., Stein D.J., Koen N., Sly P.D., Hantos Z. (2017). Determinants of early-life lung function in African infants. Thorax.

[70] Kotecha S.J., Watkins W.J., Heron J., Henderson J., Dunstan F.D., Kotecha S. (2010). Spirometric lung function in school-age children: Effect of intrauterine growth retardation and catch-up growth. Am. J. Respir. Crit. Care Med..

[71] den Dekker H.T., Sonnenschein-van der Voort A.M.M., de Jongste J.C., Anessi-Maesano I., Arshad S.H., Barros H., Beardsmore C.S., Bisgaard H., Phar S.C., Craig L. (2016). Early growth characteristics and the risk of reduced lung function and asthma: A meta-analysis of 25,000 children. J. Allergy Clin. Immunol..

[72] Green M., Mead J., Turner J.M. (1974). Variability of maximum expiratory flow-volume curves. J. Appl. Physiol..

[73] Merkus P.J., Borsboom G.J., Van Pelt W., Schrader P.C., Van Houwelingen H.C., Kerrebijn K.F., Quanjer P.H. (1993). Growth of airways and air spaces in teenagers is related to sex but not to symptoms. J. Appl. Physiol..

[74] Yammine S., Schmidt A., Sutter O., Fouzas S., Singer F., Frey U., Latzin P. (2016). Functional evidence for continued alveolarisation in former preterms at school age?. Eur. Respir. J..

[75] Narayanan M., Beardsmore C.S., Owers-Bradley J., Dogaru C.M., Mada M., Ball I., Garipov R.R., Kuehni C.E., Spycher B.D., Silverman M. (2013). Catch-up Alveolarization in Ex-Preterm Children. Evidence from 3He Magnetic Resonance. Am. J. Respir. Crit. Care Med..

[76] Forno E., Weiner D.J., Mullen J., Sawicki G., Kurland G., Han Y.Y., Cloutier M.M., Canino G., Weiss S.T., Litonjua A.A., Celedón J.C. (2017). Obesity and Airway Dysanapsis in Children with and without Asthma. Am. J. Respir. Crit. Care Med..

[77] Saad N.J., Patel J., Burney P., Minelli C. (2017). Birth Weight and Lung Function in Adulthood: A Systematic Review and Meta-analysis. Ann. Am. Thorac. Soc..

[78] Baumann S., Godtfredsen N.S., Lange P., Pisinger C. (2015). The impact of birth weight on the level of lung function and lung function decline in the general adult population. The Inter99 study. Respir. Med..

[79] Canoy D., Pekkanen J., Elliott P., Pouta A., Laitinen J., Hartikainen A.-L., Zitting P., Patel S., Little M.P., Järvelin M.-R. (2007). Early growth and adult respiratory function in men and women followed from the fetal period to adulthood. Thorax.

[80] Lange P., Celli B., Agustí A., Boje Jensen G., Divo M., Faner R., Guerra S., Marott J.L., Martinez F.D., Martinez-Camblor P. (2015). Lung-Function Trajectories Leading to Chronic Obstructive Pulmonary Disease. N. Engl. J. Med..

[81] Lopuhaä C.E., Roseboom T.J., Osmond C., Barker D.J., Ravelli A.C., Bleker O.P., van der Zee J.S., van der Meulen J.H. (2000). Atopy, lung function, and obstructive airways disease after prenatal exposure to famine. Thorax.

[82] Gaultier C. (1991). Malnutrition and lung growth. Pediatr. Pulmonol..

[83] Massaro G.D., McCoy L., Massaro D. (1988). Postnatal undernutrition slows development of bronchiolar epithelium in rats. Am. J. Physiol..

[84] Mataloun M.M.G.B., Rebello C.M., Mascaretti R.S., Dohlnikoff M., Leone C.R. (2006). Pulmonary responses to nutritional restriction and hyperoxia in premature rabbits. J. Pediatr..

[85] Mataloun M.M.G.B., Leone C.R., Mascaretti R.S., Dohlnikoff M., Rebello C.M. (2009). Effect of postnatal malnutrition on hyperoxia-induced newborn lung development. Braz. J. Med. Biol. Res. Rev. Bras. Pesqui. Medicas E Biol..

[86] Matecki S., Py G., Lambert K., Peyreigne C., Mercier J., Prefaut C., Ramonatxo M. (2002). Effect of prolonged undernutrition on rat diaphragm mitochondrial respiration. Am. J. Respir. Cell Mol. Biol..

[87] Poindexter B.B., Martin C.R. (2015). Impact of Nutrition on Bronchopulmonary Dysplasia. Clin. Perinatol..

[88] Oh W., Poindexter B.B., Perritt R., Lemons J.A., Bauer C.R., Ehrenkranz R.A., Stoll B.J., Poole K., Wright L.L. (2005). Neonatal Research Network Association between fluid intake and weight loss during the first ten days of life and risk of bronchopulmonary dysplasia in extremely low birth weight infants. J. Pediatr..

[89] Bell E.F., Acarregui M.J. (2014). Restricted versus liberal water intake for preventing morbidity and mortality in preterm infants. Cochrane Database Syst. Rev..

[90] Huysman W.A., de Ridder M., de Bruin N.C., van Helmond G., Terpstra N., Goudoever J.B.V., Sauer P.J.J. (2003). Growth and body composition in preterm infants with bronchopulmonary dysplasia. Arch. Dis. Child. Fetal Neonatal Ed..

[91] Hartnoll G., Betremieux P., Modi N. (2000). Randomised controlled trial of postnatal sodium supplementation on oxygen dependency and body weight in 25-30 week gestational age infants. Arch. Dis. Child. Fetal Neonatal Ed..

[92] Dogaru C.M., Nyffenegger D., Pescatore A.M., Spycher B.D., Kuehni C.E. (2014). Breastfeeding and childhood asthma: Systematic review and meta-analysis. Am. J. Epidemiol..

[93] Duijts L., Ramadhani M.K., Moll H.A. (2009). Breastfeeding protects against infectious diseases during infancy in industrialized countries. A systematic review. Matern. Child. Nutr..

[94] Ladomenou F., Moschandreas J., Kafatos A., Tselentis Y., Galanakis E. (2010). Protective effect of exclusive breastfeeding against infections during infancy: A prospective study. Arch. Dis. Child..

[95] Munblit D., Peroni D.G., Boix-Amorós A., Hsu P.S., Van’t Land B., Gay M.C.L., Kolotilina A., Skevaki C., Boyle R.J., Collado M.C. (2017). Human Milk and Allergic Diseases: An Unsolved Puzzle. Nutrients.

[96] Lodge C.J., Tan D.J., Lau M.X.Z., Dai X., Tham R., Lowe A.J., Bowatte G., Allen K.J., Dharmage S.C. (2015). Breastfeeding and asthma and allergies: A systematic review and meta-analysis. Acta Paediatr..

[97] Waidyatillake N.T., Allen K.J., Lodge C.J., Dharmage S.C., Abramson M.J., Simpson J.A., Lowe A.J. (2013). The impact of breastfeeding on lung development and function: A systematic review. Expert Rev. Clin. Immunol..

[98] Ogbuanu I.U., Karmaus W., Arshad S.H., Kurukulaaratchy R.J., Ewart S. (2009). Effect of breastfeeding duration on lung function at age 10 years: A prospective birth cohort study. Thorax.

[99] Soto-Ramírez N., Alexander M., Karmaus W., Yousefi M., Zhang H., Kurukulaaratchy R.J., Raza A., Mitchell F., Ewart S., Arshad S.H. (2012). Breastfeeding is associated with increased lung function at 18 years of age: A cohort study. Eur. Respir. J..

[100] Guilbert T.W., Stern D.A., Morgan W.J., Martinez F.D., Wright A.L. (2007). Effect of breastfeeding on lung function in childhood and modulation by maternal asthma and atopy. Am. J. Respir. Crit. Care Med..

[101] Dogaru C.M., Narayanan M., Spycher B.D., Pescatore A.M., Owers-Bradley J., Beardsmore C.S., Silverman M., Kuehni C.E. (2015). Breastfeeding, lung volumes and alveolar size at school-age. BMJ Open Respir. Res..

[102] Kull I., Melen E., Alm J., Hallberg J., Svartengren M., van Hage M., Pershagen G., Wickman M., Bergström A. (2010). Breast-feeding in relation to asthma, lung function, and sensitization in young schoolchildren. J. Allergy Clin. Immunol..

[103] van Meel E.R., de Jong M., Elbert N.J., den Dekker H.T., Reiss I.K., de Jongste J.C., Jaddoe V.W.V., Duijts L. (2017). Duration and exclusiveness of breastfeeding and school-age lung function and asthma. Ann. Allergy Asthma Immunol..

[104] Oddy W.H., Halonen M., Martinez F.D., Lohman I.C., Stern D.A., Kurzius-Spencer M., Guerra S., Wright A.L. (2003). TGF-beta in human milk is associated with wheeze in infancy. J. Allergy Clin. Immunol..

[105] Verduci E., Banderali G., Barberi S., Radaelli G., Lops A., Betti F., Riva E., Giovannini M. (2014). Epigenetic effects of human breast milk. Nutrients.

[106] Lelijveld N., Kerac M., Seal A., Chimwezi E., Wells J.C., Heyderman R.S., Nyirenda M.J., Stocks J., Kirkby J. (2017). Long-term effects of severe acute malnutrition on lung function in Malawian children: A cohort study. Eur. Respir. J..

[107] Korten I., Usemann J., Latzin P. (2017). “Lung sparing growth”: Is the lung not affected by malnutrition?. Eur. Respir. J..

[108] Black R.E. (2001). Micronutrients in pregnancy. Br. J. Nutr..

[109] Hovdenak N., Haram K. (2012). Influence of mineral and vitamin supplements on pregnancy outcome. Eur. J. Obstet. Gynecol. Reprod. Biol..

[110] Black R.E., Victora C.G., Walker S.P., Bhutta Z.A., Christian P., de Onis M., Ezzati M., Grantham-McGregor S., Katz J., Martorell R. (2013). Maternal and child undernutrition and overweight in low-income and middle-income countries. Lancet.

[111] Darnton-Hill I., Mkparu U.C. (2015). Micronutrients in pregnancy in low- and middle-income countries. Nutrients.

[112] Malpel S., Mendelsohn C., Cardoso W.V. (2000). Regulation of retinoic acid signaling during lung morphogenesis. Dev. Camb. Engl..

[113] Chazaud C., Dollé P., Rossant J., Mollard R. (2003). Retinoic acid signaling regulates murine bronchial tubule formation. Mech. Dev..

[114] Massaro D., Massaro G.D. (2003). Retinoids, alveolus formation, and alveolar deficiency: Clinical implications. Am. J. Respir. Cell Mol. Biol..

[115] McGowan S., Jackson S.K., Jenkins-Moore M., Dai H.H., Chambon P., Snyder J.M. (2000). Mice bearing deletions of retinoic acid receptors demonstrate reduced lung elastin and alveolar numbers. Am. J. Respir. Cell Mol. Biol..

[116] Chailley-Heu B., Chelly N., Lelièvre-Pégorier M., Barlier-Mur A.M., Merlet-Bénichou C., Bourbon J.R. (1999). Mild vitamin A deficiency delays fetal lung maturation in the rat. Am. J. Respir. Cell Mol. Biol..

[117] Barber T., Esteban-Pretel G., Marín M.P., Timoneda J. (2014). Vitamin a deficiency and alterations in the extracellular matrix. Nutrients.

[118] McGowan S.E., Doro M.M., Jackson S.K. (1997). Endogenous retinoids increase perinatal elastin gene expression in rat lung fibroblasts and fetal explants. Am. J. Physiol..

[119] Wei H., Huang H.-M., Li T.-Y., Qu P., Liu Y.-X., Chen J. (2009). Marginal vitamin A deficiency affects lung maturation in rats from prenatal to adult stage. J. Nutr. Sci. Vitaminol..

[120] Esteban-Pretel G., Marín M.P., Renau-Piqueras J., Barber T., Timoneda J. (2010). Vitamin A deficiency alters rat lung alveolar basement membrane: Reversibility by retinoic acid. J. Nutr. Biochem..

[121] Tyson J.E., Wright L.L., Oh W., Kennedy K.A., Mele L., Ehrenkranz R.A., Stoll B.J., Lemons J.A., Stevenson D.K., Bauer C.R. (1999). Vitamin A Supplementation for Extremely-Low-Birth-Weight Infants. N. Engl. J. Med..

[122] Darlow B.A., Graham P.J., Rojas-Reyes M.X. (2016). Vitamin A supplementation to prevent mortality and short- and long-term morbidity in very low birth weight infants. Cochrane Database Syst. Rev..

[123] Ambalavanan N., Tyson J.E., Kennedy K.A., Hansen N.I., Vohr B.R., Wright L.L., Carlo W.A. (2005). Vitamin A Supplementation for Extremely Low Birth Weight Infants: Outcome at 18 to 22 Months. Pediatrics.

[124] Checkley W., West K.P., Wise R.A., Baldwin M.R., Wu L., LeClerq S.C., Christian P., Katz J., Tielsch J.M., Khatry S. (2010). Maternal vitamin A supplementation and lung function in offspring. N. Engl. J. Med..

[125] Nguyen M., Trubert C.L., Rizk-Rabin M., Rehan V.K., Besançon F., Cayre Y.E., Garabédian M. (2004). 1,25-Dihydroxyvitamin D3 and fetal lung maturation: Immunogold detection of VDR expression in pneumocytes type II cells and effect on fructose 1,6 bisphosphatase. J. Steroid Biochem. Mol. Biol..

[126] Marin L., Dufour M.E., Tordet C., Nguyen M. (1990). 1,25(OH)2D3 stimulates phospholipid biosynthesis and surfactant release in fetal rat lung explants. Biol. Neonate.

[127] Sakurai R., Shin E., Fonseca S., Sakurai T., Litonjua A.A., Weiss S.T., Torday J.S., Rehan V.K. (2009). 1alpha,25(OH)2D3 and its 3-epimer promote rat lung alveolar epithelial-mesenchymal interactions and inhibit lipofibroblast apoptosis. Am. J. Physiol. Lung Cell. Mol. Physiol..

[128] Yurt M., Liu J., Sakurai R., Gong M., Husain S.M., Siddiqui M.A., Husain M., Villarreal P., Akcay F., Torday J.S. (2014). Vitamin D supplementation blocks pulmonary structural and functional changes in a rat model of perinatal vitamin D deficiency. Am. J. Physiol. Lung Cell. Mol. Physiol..

[129] Zosky G.R., Berry L.J., Elliot J.G., James A.L., Gorman S., Hart P.H. (2011). Vitamin D deficiency causes deficits in lung function and alters lung structure. Am. J. Respir. Crit. Care Med..

[130] Kho A.T., Sharma S., Qiu W., Gaedigk R., Klanderman B., Niu S., Anderson C., Leeder J.S., Weiss S.T., Tantisira K.G. (2013). Vitamin D related genes in lung development and asthma pathogenesis. BMC Med. Genom..

[131] Zosky G.R., Hart P.H., Whitehouse A.J.O., Kusel M.M., Ang W., Foong R.E., Chen L., Holt P.G., Sly P.D., Hall G.L. (2014). Vitamin D deficiency at 16 to 20 weeks’ gestation is associated with impaired lung function and asthma at 6 years of age. Ann. Am. Thorac. Soc..

[132] Gazibara T., den Dekker H.T., de Jongste J.C., McGrath J.J., Eyles D.W., Burne T.H., Reiss I.K., Franco O.H., Tiemeier H., Jaddoe V.W.V. (2016). Associations of maternal and fetal 25-hydroxyvitamin D levels with childhood lung function and asthma: The Generation R Study. Clin. Exp. Allergy J. Br. Soc. Allergy Clin. Immunol..

[133] Pacheco-González R.M., García-Marcos L., Morales E. (2018). Prenatal vitamin D status and respiratory and allergic outcomes in childhood: A meta-analysis of observational studies. Pediatr. Allergy Immunol..

[134] Litonjua A.A., Carey V.J., Laranjo N., Harshfield B.J., McElrath T.F., O’Connor G.T., Sandel M., Iverson R.E., Lee-Paritz A., Strunk R.C. (2016). Effect of Prenatal Supplementation With Vitamin D on Asthma or Recurrent Wheezing in Offspring by Age 3 Years: The VDAART Randomized Clinical Trial. JAMA.

[135] Chawes B.L., Bønnelykke K., Stokholm J., Vissing N.H., Bjarnadóttir E., Schoos A.-M.M., Wolsk H.M., Pedersen T.M., Vinding R.K., Thorsteinsdóttir S. (2016). Effect of Vitamin D3 Supplementation During Pregnancy on Risk of Persistent Wheeze in the Offspring: A Randomized Clinical Trial. JAMA.

[136] Wolsk H.M., Chawes B.L., Litonjua A.A., Hollis B.W., Waage J., Stokholm J., Bønnelykke K., Bisgaard H., Weiss S.T. (2017). Prenatal vitamin D supplementation reduces risk of asthma/recurrent wheeze in early childhood: A combined analysis of two randomized controlled trials. PLoS ONE.

[137] Roth D.E., Leung M., Mesfin E., Qamar H., Watterworth J., Papp E. (2017). Vitamin D supplementation during pregnancy: State of the evidence from a systematic review of randomised trials. BMJ.

[138] Scholl T.O., Chen X., Sims M., Stein T.P. (2006). Vitamin E: Maternal concentrations are associated with fetal growth. Am. J. Clin. Nutr..

[139] Turner S.W., Campbell D., Smith N., Craig L.C.A., McNeill G., Forbes S.H., Harbour P.J., Seaton A., Helms P.J., Devereux G.S. (2010). Associations between fetal size, maternal {alpha}-tocopherol and childhood asthma. Thorax.

[140] Beckhaus A.A., Garcia-Marcos L., Forno E., Pacheco-Gonzalez R.M., Celedón J.C., Castro-Rodriguez J.A. (2015). Maternal nutrition during pregnancy and risk of asthma, wheeze, and atopic diseases during childhood: A systematic review and meta-analysis. Allergy.

[141] Sabat R., Guthmann F., Rüstow B. (2008). Formation of reactive oxygen species in lung alveolar cells: Effect of vitamin E deficiency. Lung.

[142] Falciglia H.S., Johnson J.R., Sullivan J., Hall C.F., Miller J.D., Riechmann G.C., Falciglia G.A. (2003). Role of antioxidant nutrients and lipid peroxidation in premature infants with respiratory distress syndrome and bronchopulmonary dysplasia. Am. J. Perinatol..

[143] Falciglia H.S., Ginn-Pease M.E., Falciglia G.A., Lubin A.H., Frank D.J., Chang W. (1988). Vitamin E and selenium levels of premature infants with severe respiratory distress syndrome and bronchopulmonary dysplasia. J. Pediatr. Perinat. Nutr..

[144] Stone C.A., McEvoy C.T., Aschner J.L., Kirk A., Rosas-Salazar C., Cook-Mills J.M., Moore P.E., Walsh W.F., Hartert T.V. (2018). Update on Vitamin E and Its Potential Role in Preventing or Treating Bronchopulmonary Dysplasia. Neonatology.

[145] Brion L.P., Bell E.F., Raghuveer T.S. (2003). Vitamin E supplementation for prevention of morbidity and mortality in preterm infants. Cochrane Database Syst. Rev..

[146] Kim H.Y., Picciano M.F., Wallig M.A., Milner J.A. (1991). The role of selenium nutrition in the development of neonatal rat lung. Pediatr. Res..

[147] Baïz N., Chastang J., Ibanez G., Annesi-Maesano I. (2016). Prenatal exposure to selenium may protect against wheezing in children by the age of 3. Immun. Inflamm. Dis..

[148] Darlow B.A., Inder T.E., Graham P.J., Sluis K.B., Malpas T.J., Taylor B.J., Winterbourn C.C. (1995). The relationship of selenium status to respiratory outcome in the very low birth weight infant. Pediatrics.

[149] Darlow B.A., Austin N.C. (2003). Selenium supplementation to prevent short-term morbidity in preterm neonates. Cochrane Database Syst. Rev..

[150] Vojnik C., Hurley L.S. (1977). Abnormal prenatal lung development resulting from maternal zinc deficiency in rats. J. Nutr..

[151] Litonjua A.A., Rifas-Shiman S.L., Ly N.P., Tantisira K.G., Rich-Edwards J.W., Camargo C.A., Weiss S.T., Gillman M.W., Gold D.R. (2006). Maternal antioxidant intake in pregnancy and wheezing illnesses in children at 2 y of age. Am. J. Clin. Nutr..

[152] Miyake Y., Sasaki S., Tanaka K., Hirota Y. (2010). Consumption of vegetables, fruit, and antioxidants during pregnancy and wheeze and eczema in infants. Allergy.

[153] West C.E., Dunstan J., McCarthy S., Metcalfe J., D’Vaz N., Meldrum S., Oddy W.H., Tulic M.K., Prescott S.L. (2012). Associations between maternal antioxidant intakes in pregnancy and infant allergic outcomes. Nutrients.

[154] Martindale S., McNeill G., Devereux G., Campbell D., Russell G., Seaton A. (2005). Antioxidant intake in pregnancy in relation to wheeze and eczema in the first two years of life. Am. J. Respir. Crit. Care Med..

[155] Nwaru B.I., Erkkola M., Ahonen S., Kaila M., Kronberg-Kippilä C., Ilonen J., Simell O., Knip M., Veijola R., Virtanen S.M. (2011). Intake of antioxidants during pregnancy and the risk of allergies and asthma in the offspring. Eur. J. Clin. Nutr..

[156] Calder P.C. (2010). Omega-3 Fatty Acids and Inflammatory Processes. Nutrients.

[157] Blanco P.G., Freedman S.D., Lopez M.C., Ollero M., Comen E., Laposata M., Alvarez J.G. (2004). Oral docosahexaenoic acid given to pregnant mice increases the amount of surfactant in lung and amniotic fluid in preterm fetuses. Am. J. Obstet. Gynecol..

[158] Tenorio-Lopes L., Baldy C., Jochmans-Lemoine A., Mercier O., Pothier-Piccinin O., Seaborn T., Joseph V., Marc I., Kinkead R. (2017). Consequences of maternal omega-3 polyunsaturated fatty acid supplementation on respiratory function in rat pups. J. Physiol..

[159] Joss-Moore L.A., Wang Y., Baack M.L., Yao J., Norris A.W., Yu X., Callaway C.W., McKnight R.A., Albertine K.H., Lane R.H. (2010). IUGR decreases PPARγ and SETD8 Expression in neonatal rat lung and these effects are ameliorated by maternal DHA supplementation. Early Hum. Dev..

[160] Velten M., Britt R.D., Heyob K.M., Tipple T.E., Rogers L.K. (2014). Maternal dietary docosahexaenoic acid supplementation attenuates fetal growth restriction and enhances pulmonary function in a newborn mouse model of perinatal inflammation. J. Nutr..

[161] Rogers L.K., Valentine C.J., Pennell M., Velten M., Britt R.D., Dingess K., Zhao X., Welty S.E., Tipple T.E. (2011). Maternal docosahexaenoic acid supplementation decreases lung inflammation in hyperoxia-exposed newborn mice. J. Nutr..

[162] Ali M., Heyob K.M., Velten M., Tipple T.E., Rogers L.K. (2015). DHA suppresses chronic apoptosis in the lung caused by perinatal inflammation. Am. J. Physiol. Lung Cell. Mol. Physiol..

[163] Ma L., Li N., Liu X., Shaw L., Li Calzi S., Grant M.B., Neu J. (2012). Arginyl-glutamine dipeptide or docosahexaenoic acid attenuate hyperoxia-induced lung injury in neonatal mice. Nutrition.

[164] Sharma D., Nkembi A.S., Aubry E., Houeijeh A., Butruille L., Houfflin-Debarge V., Besson R., Deruelle P., Storme L. (2015). Maternal PUFA ω-3 Supplementation Prevents Neonatal Lung Injuries Induced by Hyperoxia in Newborn Rats. Int. J. Mol. Sci..

[165] Smith S.L., Rouse C.A. (2017). Docosahexaenoic acid and the preterm infant. Matern. Health Neonatol. Perinatol..

[166] Uauy R., Mena P. (2015). Long-chain polyunsaturated fatty acids supplementation in preterm infants. Curr. Opin. Pediatr..

[167] Manley B.J., Makrides M., Collins C.T., McPhee A.J., Gibson R.A., Ryan P., Sullivan T.R., Davis P.G. (2011). DINO Steering Committee High-dose docosahexaenoic acid supplementation of preterm infants: Respiratory and allergy outcomes. Pediatrics.

[168] Moltu S.J., Strømmen K., Blakstad E.W., Almaas A.N., Westerberg A.C., Brække K., Rønnestad A., Nakstad B., Berg J.P., Veierød M.B. (2013). Enhanced feeding in very-low-birth-weight infants may cause electrolyte disturbances and septicemia—A randomized, controlled trial. Clin. Nutr. Edinb. Scotl..

[169] Collins C.T., Makrides M., McPhee A.J., Sullivan T.R., Davis P.G., Thio M., Simmer K., Rajadurai V.S., Travadi J., Berry M.J. (2017). Docosahexaenoic Acid and Bronchopulmonary Dysplasia in Preterm Infants. N. Engl. J. Med..

[170] Bisgaard H., Stokholm J., Chawes B.L., Vissing N.H., Bjarnadóttir E., Schoos A.-M.M., Wolsk H.M., Pedersen T.M., Vinding R.K., Thorsteinsdóttir S. (2016). Fish Oil-Derived Fatty Acids in Pregnancy and Wheeze and Asthma in Offspring. N. Engl. J. Med..

